# Screening of *Strongyloides* infection using an ELISA test in transplant candidates

**DOI:** 10.6061/clinics/2019/e698

**Published:** 2019-05-28

**Authors:** Beatriz Toledo, Marcelo A Corral, Dirce Mary C L Meisel, Maiara Gottardi, Edson Abdala, Silvia F Costa, Lígia Camera Pierrotti, Susana A Z Lescano, Elenice M N Gonçalves, Vera L P Castilho, Pedro P Chieffi, Ronaldo C B Gryschek, Fabiana M Paula

**Affiliations:** I Laboratorio de Investigacao Medica (LIM/06 – Laboratorio de Imunopatologia da esquistossomose), Hospital das Clinicas HCFMUSP, Faculdade de Medicina, Universidade de Sao Paulo, Sao Paulo, SP, BR; II Hospital das Clinicas HCFMUSP, Faculdade de Medicina, Universidade de Sao Paulo, Sao Paulo, SP, BR; III Instituto de Medicina Tropical de Sao Paulo, Universidade de Sao Paulo, Sao Paulo, SP, BR; IV Secao de Parasitologia da Divisao de Laboratorio Central, Hospital das Clinicas HCFMUSP, Faculdade de Medicina, Universidade de Sao Paulo, Sao Paulo, SP, BR; V Faculdade de Ciencias Medicas, Santa Casa, Sao Paulo, SP, BR

**Keywords:** Heterologous Antigen, *Strongyloides Venezuelensis*, ELISA Test, Transplant Candidates

## Abstract

**OBJECTIVES:**

Hyperinfection or disseminated strongyloidiasis has been frequently reported after transplants and is related to high mortality. This study aimed to screen for strongyloidiasis using serological diagnoses in transplant candidates.

**METHODS:**

An ELISA test was performed with filariform larvae of *Strongyloides venezuelensis* as a source of antigen.

**RESULTS:**

In the serum from transplant candidates, anti-*Strongyloides* IgG antibodies were detected in 35/150 (23.3%) samples by soluble fractions in phosphate buffered saline (PBS), 31/150 (20.7%) samples by soluble fractions in Tris-HCl, 27/150 (18.0%) samples by membrane fractions in PBS and 22/150 (14.7%) samples by membrane fractions in Tris-HCl.

**CONCLUSIONS:**

The present results suggest the ELISA test, ideally using soluble fractions of filariform larvae *S. venezuelensis* in PBS, as an additional strategy for the diagnosis of strongyloidiasis in transplant candidates.

## INTRODUCTION

The intestinal nematode *Strongyloides stercoralis* is responsible for human strongyloidiasis, which is endemic in tropical and subtropical regions 1. This helminth may cause a benign asymptomatic infection but may also result in more severe complications, such as hyperinfection and disseminated disease, especially in immunocompromised patients 2. Case reports of hyperinfection or disseminated strongyloidiasis have been frequently reported after solid-organ transplants 3,4 and after transplantation of hematopoietic cells 5 and are frequently associated with high mortality. In this context, patients who are candidates for transplantation constitute an important group for *S. stercoralis* screening.

Early diagnosis and treatment of strongyloidiasis prior to transplantation are important factors in minimizing the probability of disease progression to more severe forms 6. Therefore, the provision of specific and sensitive techniques for the diagnosis of *S. stercoralis* infection in both donors and recipients has therapeutic potential, especially in endemic areas. The diagnosis of human strongyloidiasis is based on the observation of *S. stercoralis* larvae in feces, particularly by concentration or culture techniques 7. However, these parasitological techniques have low sensitivity, requiring multiple samples to reach 100% sensitivity 8. Thus, serological techniques have been used as alternative diagnostic tools, demonstrating higher sensitivity than that of parasitological methods 7. In a previous study by our group 9, IgG-ELISA using filariform larvae antigens of *Strongyloides venezuelensis* showed 90-100% sensitivity and 92.4-98.4% specificity.

In recent years, the number of transplants worldwide has increased considerably 10, implying that more transplant candidates are at a risk of infectious diseases such as strongyloidiasis, especially its severe forms. This study aimed to screen for strongyloidiasis by serological diagnosis in transplant candidates using *S. venezuelensis* as a source of antigen.

## MATERIAL AND METHODS

### Serum samples

Serum samples (n=150) were obtained at Hospital das Clínicas of Faculdade de Medicina, Universidade de São Paulo, state of São Paulo, Brazil (HC-FMUSP), from patients who signed informed consent. This study was integrated into a larger study on *S. stercoralis* infection and was approved by the Research Ethics Committee of HC-FMUSP (protocol no. 0123/10). The transplant candidates were 10-60 years of age from both genders and had underlying diseases conferring some degree of immune dysfunction. All of the patients included in the study had previously been administered parasitological tests [spontaneous sedimentation, modified Baermann 11, and agar plate culture 12]. Serum samples were collected at the time of stool sample and stored at -20°C.

### Parasites and antigenic fractions

Antigenic fractions were obtained according to well-standardized methods 9. Saline extracts of *S. venezuelensis* filariform larvae (L3) were obtained from charcoal cultures of feces from experimentally infected *Rattus norvegicus* (Wistar), protocol (CPE-IMT 2011/126). L3 were resuspended in phosphate buffered saline (PBS, 0.01 M, pH 7.2) or Tris-HCl (25 mM, pH 7.5) containing protease inhibitors (Sigma-Aldrich, St. Louis, MO, USA) and then disrupted in an ice bath using a tissue homogenizer. The suspensions were centrifuged at 12,400 g for 30 min at 4°C, and the supernatant was collected. The soluble fractions prepared in PBS and Tris-HCl were labeled “SS” and “TS,” respectively. The pellets were resuspended in 1% SDS, or in buffer (7M urea, 2 M thiourea, 2% CHAPS), and the supernatant was collected. The membrane fractions prepared separately in PBS and Tris-HCl were labeled “SM” and “TM,” respectively.

### ELISA test

As described previously 9, polystyrene microplates were coated with each of the antigenic fractions (SS, TS, SM, and TM) at concentrations of 10 μg/mL in carbonate-bicarbonate buffer (0.06 M, pH 9.6) prior to incubation overnight at 4°C. After washing with PBS containing 0.05% Tween-20 and 3% nonfat milk (PBS-TM), the microplates were incubated with serum samples diluted 1:200 PBS-TM for 45 min at 37°C. After washing with PBS-T, enzyme-conjugated goat anti-human IgG (Fc specific)-peroxidase antibody (Sigma-Aldrich, St. Louis, MO, USA) was added at a dilution of 1:30,000 in PBS-TM and incubated for 45 min at 37°C. The assay was developed by adding 3,3′, 5,5- tetramethylbenzidine (TMB) chromogen solution (Thermo Fischer Scientific, Waltham, MA, USA) for 6 min. The reaction was interrupted with 2 N H_2_SO_4_. Optical densities (ODs) were determined at 450 nm using an ELISA reader (Thermo Fischer Scientific, Waltham, MA, USA). Absorbance levels ≥0.309 for SS, ≥0.492 for TS, ≥0.381 for SM, and ≥0.394 for TM were considered positive. The cut-off values were determined by receiver operating characteristic curve analysis using negative and other parasite samples (n=72). The ELISA index (EI) was determined by the ratio OD/cut-off, and RI values >1 were considered positive.

### Statistical analysis

Statistical analysis was performed using GraphPad Prism version 5.0 (GraphPad Software, La Jolla, USA). Detection of IgG antibodies was analyzed by the Kruskal-Wallis test followed by Dunn's multiple comparison test. The concordance was carried out by analysis of the kappa coefficient (κ). Statistical significance was set at *p*<0.05.

## RESULTS

**Figure 1:**
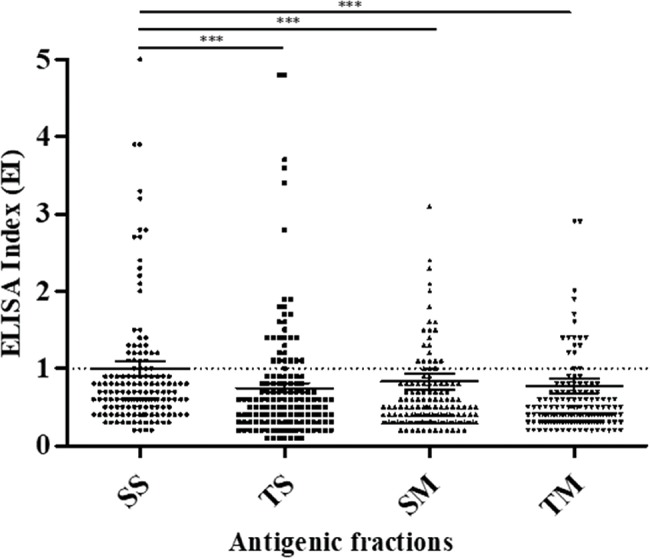
Specific anti-*Strongyloides* IgG expressed as the ELISA index (EI) for serum samples from transplant candidates using *S. venezuelensis* antigenic fractions (SS, TS, SM and TM). The dashed lines represent the positivity threshold (EI >1.0).

Anti-*Strongyloides* IgG antibodies in serum from transplant candidates were detected in 35/150 (23.3%) samples by SS, 31/150 (20.7%) samples by TS, 27/150 (18.0%) samples by SM and 22/150 (14.7%) samples by TM (Figure 1). The parasitological diagnosis of the transplant candidates showed that 15/150 (10%) of the patients' stool samples contained *S. stercoralis* larvae. Among the fifteen samples that tested positive by the parasitological methods, five showed anti-*Strongyloides* IgG antibodies detected by using SS and TS antigenic fractions, and four showed anti-*Strongyloides* IgG antibodies detected by using SM and TM antigenic fractions. Four protozoan-positive samples, including *Entamoeba coli* (n=3) and *Endolimax nana* (n=1), were observed, none of which showed reactivity in the different antigenic preparations.

When comparing the number of positive results identified using different buffers, but the same type of fraction (soluble or membrane), a moderate concordance was observed for both TS and SS (κ=0.567 (0.396–0.739)), and TM and SM (κ=0.433 (0.259–0.608)). When comparing soluble and membrane fractions within the same buffer, a moderate concordance was observed for both TS and TM (κ=0.567 (0.396–0.739)) and SS and SM (κ=0.433 (0.259–0.608)). Similarly, when the number of positive serum samples from patients was analyzed by using soluble (TS and SS) or membrane (TM and SM) fractions, kappa indices of 0.573 (0.414–0.732) and 0.781 (0.644–0.918), respectively, were observed, indicating moderate and good concordance, respectively. Comparing the results of the parasitological tests with those obtained by the ELISA, none of the antigenic fractions demonstrated superior performance.

## DISCUSSION

Currently, there is an increased concern about transplantation and *S. stercoralis* infection, especially due to the increase in the number of transplant cases 13. Camargo et al. 14 have stressed the need for new research for the rapid diagnosis and early treatment of solid-organ donors and recipients before transplantation. Several studies evaluated extracts from *Strongyloides* spp. suggesting that rodent species, such as *S. ratti* and *S. venezuelensis,* can be used as good source of antigens for the immunodiagnosis of human strongyloidiasis 15,16. This study is the first to demonstrate the detection of anti-*Strongyloides* IgG antibodies in candidates for transplantation by an ELISA test using *S. venezuelensis* as an antigen.

Although the use of serology is a challenging proposal for immunocompromised patients, many studies suggest that it may be beneficial to this profile of patients 17,18. In an ELISA using heterologous *S. ratti* antigen, 12.05% of the samples showed positive results compared with 2.4% positive results observed using parasitological techniques in immunocompromised children 19. In a study by Schaffel et al. 17 involving patients immunocompromised by malignant hematological disease, the performance of ELISA using homologous antigen (18.8% tested positive) was superior to that of parasitological methods (9.1% tested positive). In the present study, ELISA showed a higher number of positive results (14.7–20.7%) than did parasitological methods (10%), independent of the antigenic preparation. Thus, the use of the ELISA technique could be included for diagnostic screening in immunocompromised patients.

However, it is important to emphasize that antigen preparation may interfere with serological test results 1. In the present study, soluble fractions showed a higher number of positive results than did membrane fractions. Although soluble protein fractions are more frequently used in the immunodiagnosis of human strongyloidiasis 15,18, research also indicates good performance using membrane fractions 9.

The limitations of the present study may be related to the degree of immunosuppression, which may have compromised the sensitivity of the ELISA test, leading to negative ELISA results with positive parasitology. Although the ELISA test used was a noncommercial method, our results suggested its high positivity independent of the antigenic preparation.

It is therefore important to emphasize that screening should be performed with ELISA together with parasitological analysis. Considering that transplant candidates are a risk group for the occurrence of severe forms of strongyloidiasis, a positive ELISA result, independent of the antigenic preparation, is a strong indication for specific treatment. In conclusion, this study suggests the ELISA test, especially one using SS fractions, as an additional technique for the diagnosis of strongyloidiasis in transplant candidates.

## AUTHOR CONTRIBUTIONS

Paula FM, Chieffi PP and Gryschek RC conceived and designed the study. All authors participated in the performance of the study. Paula FM, Abdala E, Costa SF, Pierrotti LC, Gonçalves EM and Castilho VL participated in the recruitment and collection of patient samples. Toledo B, Gottardi M, Corral MA, Lescano SA and Meisel DM carried out the experiments. Paula FM, Chieffi PP, Corral MA and Gryschek RC were involved in the analysis and interpretation of the data. All authors contributed to revising the draft, had full access to all the data and read and approved the final version of the manuscript. Paula FM, Corral MA and Gryschek RC critically revised and completed the final draft of the manuscript.
